# Supplementary material for: Insufficient: A scoping review of structural racism and intimate partner violence in US-based immigrant communities of color
Source: PLOS Glob Public Health. 2026 Apr 29;6(4):e0006242. doi: 10.1371/journal.pgph.0006242 (PMC13127969; doi:10.1371/journal.pgph.0006242)
Supplement: S1 Table — (DOCX) [file pgph.0006242.s002.docx]

| **S1 Table: Key findings of the articles included in the scoping review** | |
| --- | --- |
| **Authors** | **Findings^a^** |
| **Tier 1: Directly attributes IPV experiences or consequences to structural racism and explicitly names structural racism as a driving factor** | |
| (Bhuyan & Velagapudi, 2013) | “The key issues that emerged from the focus group and individual interviews were consistent with common barriers that have been identified in previous research: language and interpretation, problems in dealing with the legal and criminal justice system, the mixed role of faith communities, and access to social assistance and child protection. The participants reported regional differences in immigrant women’s access to services and outcomes for immigrant victims. Depending on the context and circumstances, a resource for immigrants could equally represent a barrier or even increase a woman’s risk for deportation.” |
| **Tier 2: Does not explicitly name structural racism as a driving factor of IPV experiences or consequences, but attributes IPV experiences or consequences to structural factors of oppression that align with themes of structural racism** | |
| [(Abboud et al., 2025)](https://www.zotero.org/google-docs/?FNPzWI) | “Twelve participants reported experiencing some form of violence either from partners or parents, and ten reported witnessing it within their families. They identified multiple forms of violence within their communities and defined them as sexual, physical, and psychological violence, with the latter being the most commonly seen. Causes of IPV were attributed to individual (history of exposure to violence, age difference, personality traits, financial struggles, and lack of education on violence), relationship (family characteristics, power differences), community (lack of support system, a trauma-exposed community), and societal (divorce-related stigma, religious norms, toxic masculinity, xenophobia and Arab racism) levels factors.” |
| (Abrego & Lakhani, 2015) | “…..we demonstrate that even when they are legally present, the implementation practices of a multilayered immigration policy regime may cause them harm…As a result of “legal violence,” these legally present immigrants remain vulnerable to blocked mobility, persistent fear of deportation, and instability, confusion, and self-blame.” |
| [(Alsinai et al., 2023)](https://www.zotero.org/google-docs/?2PiuIu) | “These narratives included threats to contact authorities to interfere with an ongoing immigration process, deportation threats, and threats that would separate families. In many cases, petitioners indicated that immigration-related threats prevented them from leaving the violent partner, seeking help, or reporting the abuse. We also found mention of barriers for victims to receive protection and gain autonomy from further abuse including a lack of familiarity with US protections and laws, and restrictions on authorizations to work.” |
| (Bevilacqua et al., 2023) | “Experiences of discrimination and violence victimization were diverse in type and severity. Many women and one gender non-binary participant described experiences of intimate partner violence as well workplace violence. Men frequently described violence that occurred in public and in the workplace. Nearly all participants reported workplace discrimination. Lack of legal documentation, experiences of impunity in country of origin, and lack of knowledge of the US legal system presented barriers, while peers, social groups, and bystanders facilitated violence reporting and help-seeking.” |
| (Bhuyan, 2008) | The study finds that U.S. immigration provisions for "battered immigrants" both offer critical protections and simultaneously reinforce dominant ideologies of gender, race, and heteropatriarchy by requiring survivors to perform as "good" victims and conform to ideals of family and morality. Immigrants who cannot meet these narrow standards often "fall through the cracks" and are excluded from protections. As a result, while VAWA provisions are seen as a "bright spot" in immigration law, they discipline survivors into limited and compliant subject positions​. |
| [(Bryan et al., 2025)](https://www.zotero.org/google-docs/?0UrCTA) | “Through our descriptive analysis, perhaps unsurprisingly, we find that experiences with police range across various factors, such as gender, immigration status, and language proficiency. Though we argue that negative interactions with police may have a chilling effect on immigrant survivors and their perceptions of police and other institutions due to these intersecting identities, we do not find consistent patterns across positive or negative interactions.” |
| (Bui, 2003) | “…the study found that abused Vietnamese American women have sought help from their personal networks, the criminal justice system, and various victim service agencies. Data analyses suggest that the decisions of Vietnamese American women to reach out are complex and diverse and are shaped by various structural, cultural, and organizational factors. Acculturation on the part of abused women as well as victim services can facilitate the women’s efforts to seek help outside their personal networks.” |
| [(Chenane & Pryce, 2024)](https://www.zotero.org/google-docs/?NUlF3a) | “Our results show that participants who believe that the police practice procedural justice and distributive justice, participants who believe that a high number of African immigrant women experience intimate partner violence, and participants who believe that, compared to the police in Africa, U.S. police officers do a good job responding to intimate partner violence have a stronger sense of obligation to obey the police. In addition, participants who believe that the police are procedurally just are more willing to cooperate with the police.” |
| (Garni & Melander, 2023) | “Our ethnographic research with asylum seekers and legal advocates reveals how systems of oppression interact across borders to multiply women’s vulnerability to victimization, obstruct their access to critical resources, bar their flight and safe passage, and restrict their access to asylum.” |
| (Gezinski & Gonzalez-Pons, 2021) | “Overwhelmingly, participants communicated an immense need for emergency shelter upon exit from an abusive relationship, yet reported limited shelter space. Service providers reported that scarce funding coupled with grantor-imposed expectations impede their ability to serve all survivors in need. When turned away from shelter, survivors resorted to staying in a motel, car, homeless shelter, or even returning to the perpetrator. Barriers to obtaining permanent housing included unaffordability, landlord discrimination, and insufficient documentation. Indigenous and immigrant survivors encountered amplified barriers to housing stability. Despite the prominence of Housing First in Utah, survivors were unable to access much needed housing resources.” |
| (Giordano et al., 2021) | “Organizational access and citywide access across HIV/AIDS (mean = 1.94, *SD* = 0.83), mental health (mean = 1.37, *SD* = 0.95), substance use (mean = 1.11, *SD* = 0.74), and DV (mean = 1.49, *SD* = 0.97) services were perceived as far from optimal. Domain scores were highest for accessibility (mean = 1.66, *SD* = 1.03), followed by quality (mean = 1.44, *SD* = 0.79), availability (mean = 1.41, *SD* = .81), and adequacy (mean = 1.24, *SD* = .75).” |
| [(Gray et al., 2024)](https://www.zotero.org/google-docs/?MoRQDq) | “We find that the relationship between firearms legislation and IPH varies in magnitude and direction across specific race/ethnicity female victimization groups.” |
| [(Kaufman, 2024)](https://www.zotero.org/google-docs/?TpASNj) | “Drawing on theoretical predictions and analyses of the Add Health data, I found evidence that discrimination and unfair treatment by police had independent effects on IPV perpetration for Black and Latino men. The effect of racial injustices varied for Latino men such that both types were strong contributors to IPV perpetration for immigrant Latino men but not US born Latino men.” |
| (Ingram et al., 2010) | Immigrant women who self-petitioned under VAWA faced extensive emotional, financial, and legal barriers, including retraumatization during the application process, fear of deportation, financial insecurity due to delayed work permits, and inconsistent treatment from service providers. Positive support from trained victim advocates and legal service providers was critical in helping women navigate the process and rebuild their lives. Despite the protections VAWA offers, systemic barriers and uncertainty made accessing safety and stability a prolonged and difficult journey. |
| (Kimberg et al., 2021) | “Of 667 patients approached, 531 (80%) agreed to participate: 32% UDLI, 33% LLRC, and 35% NLRC. Of the 27.5% of respondents who knew someone who experienced DV in the past year, 46% stated that the DV victim was afraid to seek ED care; there was no significant difference in this rate between groups. The most common fears reported as barriers to disclosure were fear the doctor would report DV to police (31%) and fear that the person perpetrating DV would find out about the disclosure (30.3%).” |
| [(López‐Zerón et al., 2025)](https://www.zotero.org/google-docs/?sspspy) | “Study findings reveal multifaceted housing experiences and challenges, including unsanitary and unsafe living conditions, landlord abuse, as well as discrimination and language barriers. The concept of dignidad (dignity) was central to survivors' narratives, which survivors identified as integral to safe and stable housing.” |
| [(Marrs Fuchsel, 2024)](https://www.zotero.org/google-docs/?aFUG0R) | “Participants’ qualitative responses included an increase of intimate partner violence during the pandemic. Types of support systems included reaching out to police departments, hospitals and health-care settings, and community-based agencies. Findings indicated a 47% positive response rate when working with police officers (e.g., bilingual Spanish-English speaking police officers), and the participants reported being supported by the agency staff where they received services.” |
| (Messing et al., 2015) | “Fear of deportation was a significant predictor of Latinas’ perceptions of the procedural fairness of the criminal justice system. “ |
| (Muchow & Amuedo-Dorantes, 2020) | “We find that domestic violence calls per capita dropped in LAPD reporting districts with a higher concentration of Latino noncitizens as awareness about immigration enforcement increased.” |
| [(Nayak et al., 2023)](https://www.zotero.org/google-docs/?7BQFco) | “Findings indicated that structural discrimination, social exclusion, and dehumanization compound existing trauma and negatively impact survivors’ well-being.” |
| (Parson & Heckert, 2014) | “Women’s narratives show how biopolitical insecurities are present in familial interactions. In a sense, women’s narratives reveal how families become sites of biopolitical enforcement. Intimate partners’ threats and reminders of biopolitical insecurities intensify the interpersonal insecurities in a mutually reinforcing manner. The family becomes less and less of a safe haven due to abuses. At the same time, protections for women who suffer intimate partner violence are often channeled through the judicial system in the United States, precisely an arena where many undocumented/precariously documented women feel vulnerable. These women find themselves in a double bind, a dual layered golden cage of biopolitical and intimate insecurities. Abusers’ references to biopolitical power within intimate relationships rupture the familial trust and security that can be even more crucial for women who lack documentation or inhabit a precariously documented space.” |
| (Raj A et al., 2005) | “The odds of reporting IPV (23% of the sample)were higher for women who reported that their partners refused to change their immigration status (OR 7.8; CI 1.4, 44.6) or threatened them with deportation (OR 23.0; CI 4.5, 118.8) and for those on spousal dependent visas (OR 2.8; CI 1.1, 7.4) than they were for other women. Abused women interviewed also described how their partners used immigration laws prohibiting them from working or petitioning for status change to limit their autonomy.” |
| (Reina & Lohman, 2015) | “Our findings reveal that intersecting structural and institutional conditions make it more complicated for Latina victims to respond to partner abuse. Some of the most salient barriers include: unstable residency status, experiences of institutional discrimination, and economic inequality.” |
| (Rodriguez et al., 2018) | “All participants in this study described emotional stress, fear, and restrictions in their day-to-day life attributed to the anti-immigrant sociopolitical climate, and adults also spoke to work related stress and economic insecurity. Both adults and children described harassment by strangers, coworkers, and/or peers. With regard to DV, women tended to describe immigration stress as exasperating family conflict, while men viewed these external stressors as discouraging DV. Distrust of police and other formal supports was a key theme underlying adult and youth perceptions of help-seeking for DV. The findings of this study suggest that anti-immigrant sentiment and policy creates undue stress for Latino families and barriers to formal help-seeking for DV.” |
| [(Sabri & Campbell, 2024)](https://www.zotero.org/google-docs/?taQx7j) | “Participants shared that the partners’ possession of a firearm can increase risk for firearm related injury or fatality in situations of escalation of violence. Abused women living with a partner with firearm possession live in constant fear or threat. The presence of a firearm can also be a trigger of unwanted memories of the past and can have legal and other consequences for survivors. Impediments to reporting threats to safety were barriers such as lack of knowledge of firearm-related risks and gender and social norms.” |
| (Singh & Bullock, 2020) | “Although newspaper portrayals of all three provisions were examined, coverage overwhelmingly focused on tribal sovereignty over LGBT survivors and undocumented immigrants. Overall, we found that racial marginalization and its relationship to violence was covered superficially, that law enforcement was portrayed as the primary mechanism for protecting survivors, and that discussion of structural roots of violence was minimal. We regard this framing as a missed opportunity for raising public awareness of the intersectional nature of domestic violence. Implications for social policy and directions for future research are discussed.” |
| (Solis & Heckert, 2021) | “Both women’s narratives highlight the ways U.S. immigration laws led to a sense of entrapment in abusive relationships during their pregnancies, which put them at heightened vulnerability. Further, the stress they experienced had the potential to lead to embodied health vulnerabilities. The case studies presented illustrate the ways the law can fuel processes that contribute to health risks, highlighting how U.S. immigration law can function as legal violence.” |
| (Valdovinos et al., 2021) | “The analysis of the survivors’ testimonios also offered detailed stories to help us understand the intersectional experiences related to the survivors’ gender, ethnicity, social class, and undocumented immigration status when seeking help.” |
| [(Valdovinos & Vanegas, 2024)](https://www.zotero.org/google-docs/?12oqPP) | “Thematic analyses indicated that the parent’s immigration status restricts the entire family’s help-seeking participation. Barriers and facilitators were identified.” |
| (Yuan et al., 2022) | “We found that differences between subgroups of immigrants are reduced when procedural justice is included in the multilevel models.” |
| [(Zero et al., 2023)](https://www.zotero.org/google-docs/?7OUZiA) | “The major barriers expressed by the interviewees regarding IPV screening and disclosure include limited opportunities for IPV screening, misinformation about legal rights from abusers, fear of deportation and separation from children, and lack of knowledge about resources.” |
| ^a^Direct quotes are presented with quotation marks | |
