# Supplementary material for: Insufficient: A scoping review of structural racism and intimate partner violence in US-based immigrant communities of color
Source: PLOS Glob Public Health. 2026 Apr 29;6(4):e0006242. doi: 10.1371/journal.pgph.0006242 (PMC13127969; doi:10.1371/journal.pgph.0006242)
Supplement: Attachment — Submitted filename: Response to Reviewers 11.3.25.docx [file pgph.0006242.s004.docx]

**Dear Editorial Team,**

We are grateful for the opportunity to revise and strengthen our manuscript titled **Insufficient: A scoping review of structural racism and intimate partner violence in US-based immigrant communities of color** based on the valuable feedback from the reviewers and editors. We deeply appreciate their insightful comments, which have helped us clarify and improve the manuscript. Below, we provide a detailed, point-by-point response indicating how each comment has been addressed.

**Editor:**

1. Theoretical framing: While the manuscript introduces structural racism and intersectionality, it remains somewhat descriptive rather than analytical. There is an opportunity to deepen engagement with critical race and feminist theory:
   Integrate conceptualizations of structural racism as a relational system of power (Bailey et al., 2021; Pirtle, 2020; Krieger, 2023).

We thank the editor for the opportunity to deepen engagement with critical race and feminist theories to move beyond a descriptive approach. We have integrated conceptualizations of structural racism as relational systems of power that continuously reproduce and maintain racial hierarchies, privileging whiteness through the unequal distribution of resources, rights, and recognition (for example, lines 79-87). We have also woven more of this explicit language into our discussion.

2. Theoretical framing: Situate the analysis within feminist and decolonial frameworks (Crenshaw, Collins & Bilge, Davis, Mohanty) to emphasize how the failure to name structural racism perpetuates epistemic injustice and marginalization of immigrant women’s experiences.

We have revised the introduction to include a paragraph that more clearly outlines our theoretical framing with Crenshaw and Collins work on intersectionality and have expanded this to be situated within feminist and decolonial frameworks (Crenshaw, Collins & Bilge, Davis, Mohanty).

Lines 135-553 now read as:

“Drawing on the scholarship of Black and transnational feminists, this review is guided by intersectionality as a core analytic framework, which conceptualizes power as relational and guides an examination of how multiple interlocking systems of oppression and power, such as gender inequity, immigration systems, and structural racism, shape lived experiences. Through this framework, structural racism operates as a cross-cutting system that interacts with gendered and immigration related hierarchies to reproduce and maintain compounded inequities. A decolonial lens further extends this analysis by interrogating how Western-centric epistemologies and colonial logics shape what forms of knowledge are legitimized, whose experiences are centered, and whose are rendered invisible in IPV research. Prior scholarship suggests that when structural racism is not explicitly named as a driver of inequities, such omissions are a reflection of epistemic injustice. For example, Western-centered research paradigms rooted in colonial, gendered, and racial hierarchies privilege frameworks and data derived primarily from white women’s experiences, while overlooking or inadequately capturing the realities of women of color, particularly immigrant women. Recognizing this epistemic injustice underscores the need for this scoping review to understand how IPV scholarship has engaged with structural racism in IPV literature among US-based immigrants of color. This is an essential step toward guiding and transforming the knowledge base to better reflect the realities of immigrant women of color experiencing IPV.”

3. Methodological transparency: Clarify the decision-making process behind expanding inclusion into two tiers.
   a. How was the threshold defined between explicit and implicit references to structural racism?

To enhance methodological transparency, we elaborated on the decision-making process used to expand inclusion into two tiers and clarified how the threshold between explicit and implicit references to structural racism was defined in Lines 267-292. Initially, inclusion was limited to studies that explicitly named and attributed structural racism as a determinant of IPV. Following initial full-text review, only one study met this criterion, revealing that many studies examined structural determinants consistent with structural racism without explicitly naming or attributing it. To capture this broader conceptual scope, the research team expanded inclusion to a two-tier system.

Any explicit attribution and naming of structural racism as a determinant of IPV among U.S.-based immigrants of color was categorized as Tier 1. All implicit references and attributions of structural racism, where structural factors of oppression aligned conceptually with our definition of structural racism (see definition and search terms in Table 1, concept 2) but were not explicitly named, were categorized as Tier 2. We have revised this section to include some examples of implicit references and attributions in lines 267-292.

After redefining the inclusion criteria, the full-text review was conducted again by the same two independent reviewers using the revised tiered framework. Because the abstract review phase had already captured key study details related to the scoping review goals (e.g., population, IPV form, structural determinants), it was not repeated. Instead, the reviewers returned to the full texts to ensure that all eligible studies were reclassified appropriately under the expanded criteria (lines 267-292).

b. Was interrater reliability assessed, and how were discrepancies resolved?

Interrater reliability was not calculated, as this scoping review is guided by intersectional and decolonial frameworks. The purpose of this review was not to measure agreement across reviewers, but to ensure conceptual consistency and reflexivity in identifying how structural racism was named, attributed, and operationalized across studies. To maintain rigor, two reviewers independently screened and reviewed studies during full-text review, and met regularly to discuss discrepancies and reach consensus through iterative dialogue. Any unresolved discrepancies that remained between the two reviewers were decided on by the Senior author. This collaborative and reflexive process aligns with best practices for critical scholarship, where emphasis is placed on transparency and shared interpretation rather than statistical agreement. This process has been clarified in lines 267-292.

4. Methodological transparency: The extraction and synthesis appear to have been performed by a single reviewer. Including a brief description of quality assurance measures (e.g., verification, calibration) would strengthen credibility.

The data extraction process focused on descriptive study characteristics (e.g., study design, form of IPV examined, immigrant group, and form of structural racism addressed) rather than interpretive or thematic coding. Accordingly, the extraction was conducted using a structured table with predefined fields. The narrative synthesis focused on how studies conceptualized or operationalized structural racism, ensuring transparency and traceability across extracted data. This process has been clarified in lines 296-303..

5. Analysis and interpretation:The Tier 1/Tier 2 binary, while useful, risks oversimplifying epistemic complexity. Some Tier 2 studies may reflect structural silencing rather than conceptual absence. A brief reflection on this would enhance the critical depth of the discussion.

We have included a brief reflection on the epistemic complexity of this work and have expanded our discussion of publication bias from the limitation section into our discussion as well. Lines 398 - 404 now read: It should be noted that some Tier 2 studies may reflect structural silencing rather than conceptual absence, wherein external, institutional, or disciplinary constraints prevent researchers from explicitly naming structural racism. While this possibility cannot be fully assessed within the scope of this review, it warrants consideration in future work, particularly as publication bias and structural silencing may become increasingly salient in shaping how scholars frame structural racism as a determinant of IPV.

6. Analysis and interpretation :The results section could benefit from synthesizing differences across immigrant groups (e.g., Latinx, Asian, African, Arab) and by form of IPV or structural factor, even narratively.

The goals of this scoping review are to 1) map the extent to which structural racism is examined and named as an upstream factor impacting IPV experiences and consequences in US-based immigrant communities of color; 2) explore the depth of discussion regarding structural racism when the term was used; and 3) provide a roadmap for future work on operationalizing, naming, and attributing structural racism in IPV research focused on US-based immigrants of color. Therefore, we did not include a synthesis across immigrant groups in our results section. However, we have expanded on this issue in our discussion section.

Lines 530 – 543 now read: “It should be noted that most of the articles reviewed in the scoping review were focused on Latino immigrant populations, reflecting a systematic underrepresentation of additional immigrant populations. While synthesizing differences in the experiences of structural racism and IPV among immigrant groups goes beyond the scope of this review, many migrant populations have been shown to experience similar structural stressors. However, future research on the role of structural racism as a driver of IPV  should examine differences across immigrant groups, form of IPV, and manifestation of structural racism. As disparities are often driven not solely by ethnic origin, but also by an individual's position in the local social hierarchy, future research should examine differences within immigrant groups, as immigrants who are darker skinned or racialized as Black could face greater discrimination…”

7. Implications and future directions: The discussion should go beyond a “call to action” by offering concrete pathways:
   a. How can IPV research explicitly operationalize structural racism (e.g., indicators related to policy, enforcement, segregation, or economic exclusion)? How can national data systems and funders integrate anti-racist measures?

We have expanded our implications and future directions to offer concrete pathways throughout the discussion section. For example, lines 500 – 523 now read: Recent research has recommended novel methodological approaches for measuring structural racism. Hardeman et al. recommend measurements focused on historic, geographic, and ecological manifestations of structural racism with measures including historical prevalence of slavery, lynching, and other manifestations of racialized violence, prevalence of discriminatory federal, state, and local policies, geographic segregation, measures of redlining, and the use of latent variable approaches to measure multifaceted exposures of structural racism. Gee and Ford have identified multiple dimensions of structural racism that should be examined as fundamental causes of health disparities, including social segregation, immigration policy, historical factors, and examples of structural racism in cyberspace. Krieger recommends measures that can be categorized as “explicit” (laws, policies, and rules explicitly designed to discriminate against target groups), “non-explicit” (laws, policies, and rules designed to evade antidiscrimination laws to discriminate against target groups), or area-based or institutional non-rule measures regarding legacies and indicators of structural injustice (population-based data quantifying the disparities between the targeted group the privileged group, the prevalence of discriminatory institutional or public practices, or the presence of public monuments or commemorations supporting racism). To facilitate this research, national data systems should begin collecting data on these measures, and funders should require proposals to outline how structural racism will be conceptualized and operationalized.

b. What are the implications for practice and policy (e.g., survivor services, prevention frameworks)?

The implications for practice and policy are detailed in Table four and we have integrated this into the discussion section. Lines 515 – 523 now read: Research that explicitly operationalizes and examines measures of IPV and structural racism among US based immigrants could guide the development of programs and policy, including the development of prevention frameworks that account for stressors related to structural racism, survivor services that provide legal and immigration support to help survivors navigate discriminatory systems, and policies that aim to support immigrant survivors by directly addressing barriers to seeking help posed by structural racism.

c. Connecting findings to the US National Action Plan to End GBV could anchor the paper’s policy relevance.

In addition to our policy implications detailed in Table four, we have revised the discussion to draw more explicit connections to the US National Action Plan, should it be reestablished. Lines 185-188 now read: Such work is particularly important as immigrant communities are a priority population in the first-ever US National Action Plan to End Gender-Based Violence (GBV), necessitating research on how to best measure and address challenges faced by immigrant survivors due to structural racism

Additionally, Lines 639 – 641 now read: Such efforts are well-aligned with the US National Action Plan to End GBV and could guide future iterations of the plan by outlining the role of structural racism in IPV and the need to incorporate it as a driver in future research, policies, and programs.

8. Literature scope: Include recent works on measuring and conceptualizing structural racism in public health (e.g., Hardeman et al., 2022; Krieger et al., 2023; Gee & Ford, 2011). Expand feminist and intersectional citations to foreground the intellectual lineage of this critique.

We thank the editor for this critical suggestion and have included more recent works of these scholars throughout the manuscript.

Reviewer 1:

Reviewer #1: For this article, a scoping review of the literature was conducted to understand the impact of structural racism and IPV on immigrants of color. While the purpose of the review is important and would fulfill a gap in the literature, I find that the scoping review is underdeveloped. There are inconsistencies between the aims of the review and the review strategy for the article. Furthermore, there is confusion on whether the authors really only examined structural racism, as some of the terms are capturing other forms of racism, discrimination, and oppression. Finally, the results section seems severely underdeveloped, as the authors merely provided a broad and brief summary of the articles, instead of any examination or analysis. Though it’s a scoping review, a discussion of how the articles relate to the aims of the review, including **definitions**, **consequences**, **gaps**, etc., would strengthen this manuscript. On a smaller note, there are inconsistencies with spacing and formatting as well.

**Introduction**
In the introduction, the authors say that the immigrant health advantage erode due to systemic inequalities and cumulative impact of inequities. However, it would be helpful to **discuss the systematic inequalities and inequities that the authors are referring to in this sentence and to describe how they lead to worsen health for immigrants.**

We have included a few examples of inequities in lines 64-65. The following three paragraphs go into greater detail on the types of inequities and how they contribute to worse health.

The second paragraph of the introduction seems undeveloped. There are a bunch of ideas about systemic oppression, the clumping of diverse groups into one group called people of color, and how these all lead to health disparities. However, all together, the topics are glossed over and would be better understood by explaining these relationships further.

The paragraph beginning with, “The interplay of these intersecting identities and…” feels repetitive. It could be woven into the paragraphs before in the introduction.

We have revised the introduction for conceptual clarity. Please see our responses to the editor for further detail (# 1, 2,).

Methods
Throughout the manuscript, there seems to be differences in spacing within and between paragraphs. Please make spacing consistent throughout.

The spacing within and between paragraphs has been addressed.

How were discrepancies resolved by the team?

After a full text review using the search criteria, the authors state that only one person has met the criteria. Therefore, the authors widened the search criteria. When the authors widened the search criteria, did the authors return back to the abstract review? If not, did the authors start the full text search again? Was it blinded again? What was this process like of changing the criteria in the middle of the review?

Please see our response to the editors feedback above in #3.

In the introduction, the authors stated that the authors wanted to discuss IPV as being rooted in systemic inequalities, instead of solely being a product of systemic inequalities. However, it seems as though IPV was named as a consequence to structural factors in Tier 2. Hence, it seems that allowing articles in Tier 2 goes against one of the aims of the scoping review. Can the authors rectify this? Similarly to my previous comment, Tier 2 also seems less focused on structural racism and, rather, discuss structural barriers, structural inequalities, or structural barriers. I am struggling with the categorizing of all of the terms for structural racism, as there may be other systems of oppression, power, and discrimination at play other than racism. For example, a lot of what the authors discuss can actually be categorized as “Systemic or institutional xenophobia”.

The goal of our introduction is to frame IPV not solely as a product of individual behavior, attitude, gender or culture, but as a product of those factors, in addition to mutually reinforcing systems of oppression and power.

The reviewer is correct that Tier 2 captures articles that discuss structural barriers and structural inequalities. This distinction is central to our tiered framework. As clarified in Table 1 and the Methods section, Tier 2 captures studies that attribute IPV experiences or consequences to structural factors, such as economic marginalization, immigration enforcement, or systemic disinvestment, that conceptually align with structural racism but do not explicitly name it as such. The reviewer is also correct that these studies may be categorized as examining “systemic or institutional xenophobia or other intersecting systems of oppression” which is why they are in Tier 2, as they do not call it structural or systemic racism.

However, the absence of explicit naming of structural racism is precisely the epistemic pattern of violence this review seeks to highlight. The conceptual overlap between xenophobia, immigration policy, and racialization processes illustrates how structural racism operates through multiple, intersecting systems of oppression. And failing to name and attribute these mechanisms as structural racism perpetuates epistemic injustice and the marginalization of immigrant women’s experiences.

We have revised the introduction to clarify these concepts throughout the introduction.

Table 3 is unreadable and cut off for me. It would be better if it was turned so that we can see the entire table. However, in this table, it would be helpful to specify the information that the articles did provide for the important attribute. Otherwise, it is difficult to understand how the authors assessed fit if, for example, key attributes weren’t specifically discussed.

The table has been revised so that it is readable and no longer cut off. The table includes the key attributes that were used to assess fit outlined in our inclusion criteria. For example, immigrant group, form of IPV, form of structural racism, etc.

It would be helpful for the authors to explain “narrative synthesis.” Also, it would be helpful for the authors to describe why it was suitable for this scoping review.

We have revised the methods section to expand on this. Lines 296 - 299 now read: Narrative synthesis is an approach of synthesizing findings from multiple studies that relies on textual analysis of a study and is well-suited for analysis that requires a nuanced analysis of how complex factors such as structural racism are operationalized in the study [72].

Results
I am a little confused why an article was not included because it discussed another type of racism, even though the authors’ definition and operationalization of structural racism is broad and have included terms like ‘police brutality’, which could easily be categorized as racial profiling.

I am a bit confused about how fear of being deported or of deportation is a theme of structural racism.

We thank the reviewer for this comment and the opportunity to clarify our conceptual framing. Our review used Bailey et al.’s (2021) definition of structural racism as “the totality of ways in which societies foster racial discrimination through mutually reinforcing systems such as housing, education, employment, criminal justice, and immigration.” Under this definition, structural racism encompasses both explicit racial discrimination (e.g., racial profiling or police brutality) and policies or systems that racialize and marginalize populations, even when race is not overtly named. Please refer to table 4 to see the range of search terms used to capture manifestations of structural racism.

For this reason, fear of deportation and immigration enforcement are interpreted as manifestations of structural racism within immigrant communities of color. Immigration laws and practices in the U.S. have historically been racialized, granting and denying entry, citizenship, and protection along racial and ethnic lines. These systems differentially expose immigrants of color to criminalization, surveillance, and social exclusion, reflecting the same structural logics that underpin other racialized institutions such as policing and housing.

The articles excluded for ‘other forms of racism’ did not align with concept 2 of our search terms and approach focused on structural, systemic, and institutional racism. For example, articles that focused on interpersonal racism were excluded

We have included an example of how fear of deportation is a theme of structural racism. Lines 101 - 107 now feature an explanation of how U.S. immigration policy and enforcement were designed, and continue to perpetuate structural racism targeting specific racial categories created by U.S. immigration law in relation to whiteness. This should show that the targeting of specific “non-white” groups indicates that the systemic issues are racially motivated in a way that goes beyond general xenophobia.

For tier 1: The authors state that the article discusses intersectionality with an acknowledgement to structural racism, which tells me that structural racism was not the main focus of the article. Again, this is confusing as the purpose of the scoping review lead me to believe that structural racism should have been one of the main foci of the articles included in the review.

In the study referenced, intersectionality was used as a framework to examine interlocking systems of oppression and power, such as immigration, gender inequity, and structural racism, that shape IPV experiences and outcomes. Within this framework, structural racism was explicitly named and attributed as one of the systems driving IPV in immigrant communities. Therefore, because it explicitly named and attributed structural racism as a determinant of IPV in immigrant communities, it met the inclusion criteria for Tier 1.

The description of the Tier 1 study has been updated to clarify how it was emphasizing the dominant role of structural racism as a barrier for seeking IPV-related services and support. Lines 347-348 now read: By addressing the systemic barriers and inequities faced by these women and the explicit role played by structural racism as a significant barrier for seeking support, the paper acknowledged the role of structural racism and also provided a comprehensive analysis of its impact on IPV and the strategies advocates use to support affected individuals [[46]](https://www.zotero.org/google-docs/?MajeVa).

The results section is severely lacking. It does not integrate the articles and merely summarizes across the articles very broadly. A deeper discussion of gaps, findings, key definitions, differences by immigrant communities, and other key findings that would extend the literature. The authors did not critically examine the articles. The scoping review seems incomplete.

We would like to reiterate that the purpose of our scoping review and related findings is not to summarize or synthesize the empirical findings of included studies. Rather, our objective is to systematically map how structural racism is named, conceptualized, and attributed as an exposure or determinant of IPV within the literature on immigrant communities of color in the U.S. Our data extraction and analysis were therefore designed to prioritize how structural racism is discussed in relation to IPV, rather than the specific outcomes, effectiveness, or quality of individual studies and are well aligned with our goals for this scoping review outlined in the last paragraph of our introduction. This is a similar approach taken by other scholars in the public health field, for example: Hardeman RR, Murphy KA, Karbeah J, Kozhimannil KB. Naming Institutionalized Racism in the Public Health Literature: A Systematic Literature Review. Public Health Reports®. 2018;133(3):240-249. doi:[10.1177/0033354918760574](https://doi.org/10.1177/0033354918760574)

Discussion
The first sentence of the discussion says this is “one of the first reviews to examine….”. However, I do not believe the articles were critically examined.

We would like to reiterate that the purpose of our scoping review and related findings is not to summarize or synthesize the empirical findings of included studies. Rather, our objective is to systematically map how structural racism is named, conceptualized, and attributed as an exposure or determinant of IPV within the literature on immigrant communities of color in the U.S. Our data extraction and analysis were therefore designed to prioritize how structural racism is discussed in relation to IPV, rather than the specific outcomes, effectiveness, or quality of individual studies and are well aligned with our goals for this scoping review outlined in the last paragraph of our introduction. This is a similar approach taken by other scholars in the public health field, for example: Hardeman RR, Murphy KA, Karbeah J, Kozhimannil KB. Naming Institutionalized Racism in the Public Health Literature: A Systematic Literature Review. Public Health Reports®. 2018;133(3):240-249. doi:[10.1177/0033354918760574](https://doi.org/10.1177/0033354918760574)

The discussion is the most thorough part of the manuscript, with rich information about recommendations.

Reviewer 2:

Reviewer #2: Introduction
• The definition of structural racism (lines 76–83) could be sharpened to better distinguish it from related terms such as systemic and institutional racism, given the manuscript’s emphasis on conceptual clarity.

We thank the reviewer for this thoughtful comment. We draw on Bailey et al.’s (2021) definition of structural racism, which is intentionally broad and encompasses systemic and institutional forms of racism. Bailey et al.’s definition varies from how Braverman et al distinguish systemic and structural racism. Accordingly, we did not distinguish between these terms for the purposes of this review, as noted in the Methods section. To enhance conceptual clarity, we have now added this clarification to the Introduction as well. Please also refer to our responses to the editors feedback in # 1 and 2.

• The focus on male-perpetrated IPV against women is justified but somewhat restrictive; the authors should explicitly acknowledge exclusion of other gender identities and same-sex IPV to avoid reinforcing a heteronormative framing.

We have expanded on our notation of this as a limitation, to be included when contextualizing our results. For example, lines 624 - 626 now read: Importantly, while this review focused on male-perpetrated IPV against women and girls due to its disproportionate impact on immigrant women, other forms of IPV, including same-sex IPV and IPV perpetrated against other gender identities, could also be driven in part by structural racism.

Methods
• The criteria distinguishing Tier 1 and Tier 2 could be explained more precisely. For example, were articles that used “institutional racism” or “systemic racism” but not “structural racism” considered Tier 1 or Tier 2?

We have revised our definition of structural racism more clearly in the introduction. Please see our responses to the editor's feedback in # 1 and 2.

• The Methods section would benefit from a decision tree or supplemental table showing how terms like “legal violence” or “structural violence” were coded relative to structural racism.

Please see our response to the editor's feedback above in # 3 a.

• Tables 2 and 3 are very dense; moving detailed study characteristics to the appendix and keeping a summary in-text would improve readability.

Thank you for this suggestion. We have received conflicting suggestions on this table, which are all important to consider. We are happy to create an abridged table and are keen to hear the editor's thoughts on this.

Results
• Some numbers in text vs. PRISMA figure may need reconciliation for consistency. Please review the numbers

We thank the reviewer for catching these inconsistencies. We have reconciled the PRISMA and text for consistency.

• The disproportionate focus on Latina/o groups should be emphasized more strongly as a structural issue in the literature itself (i.e., systemic underrepresentation of African, Arab, and Asian immigrant populations).

Please see our response to the editor's feedback in # 6.

Discussion
• Recommendations could be made more actionable. For example, suggest specific measures (e.g., redlining indices, immigration enforcement intensity, housing discrimination data) that could be linked to IPV outcomes.

Please see our response to the editor’s feedback in # 7.

• The discussion of community-engaged research would be stronger if linked back to specific included studies that exemplified this approach.

We thank the reviewer for their suggestion. Lines 562 - 566 now read: Importantly, our findings noted that few studies explicitly discussed community engagement in their work. We identified 5 studies that used community-based participatory research or participatory action research study designs that could provide greater insight into the topic while enabling research participants to contribute to the design of research that addresses needs they observe in their own communities [42,47,54,57,64].

• Limitations section is appropriate, but the authors should expand on the potential publication bias (studies that name racism may face higher barriers to publication).

We have expanded on the potential of publication bias, with the additions in bold. Lines 398-404 now read as:

Lastly, there may be potential publication bias, where studies that explicitly name or address structural racism might be less likely to be published [123,124], potentially skewing the review’s findings on articles explicitly naming structural racism in IPV literature. **Publication bias that results in studies that name structural racism facing higher barriers to publication could play an unmeasured role in explaining the gap in IPV literature, examining how structural racism shapes IPV vulnerability.**
