# Supplementary material for: Insufficient: A scoping review of structural racism and intimate partner violence in US-based immigrant communities of color
Source: PLOS Glob Public Health. 2026 Apr 29;6(4):e0006242. doi: 10.1371/journal.pgph.0006242 (PMC13127969; doi:10.1371/journal.pgph.0006242)
Supplement: Attachment — Submitted filename: Point by point letter 2.19.26.docx [file pgph.0006242.s005.docx]

**Dear Editorial Team,**

We are grateful for the opportunity to revise and strengthen our manuscript titled **Insufficient: A scoping review of structural racism and intimate partner violence in US-based immigrant communities of color** based on the valuable feedback from the reviewers and editors. We deeply appreciate their insightful comments, which have helped us clarify and improve the manuscript. Below, we provide a detailed, point-by-point response indicating how each comment has been addressed.

1. Please add a concise summary of the tiering decision rules (in an in-text box or supplement) clarifying the criteria for (a) explicit naming, (b) attribution, and (c) implicit alignment terms (such as legal violence or structural violence). This addition will enhance reproducibility for borderline classifications.

**Summary of Tier Categorization**

| **Article Tier Classification** | **Criteria** |
| --- | --- |
| **Tier 1** | a) Explicitly names structural racism as a driving factor of IPV experiences or consequences;   ***and***   b) Directly attributes IPV experiences or consequences to structural racism. |
| **Tier 2** | a) Does not explicitly name structural racism as a driving factor of IPV experiences or consequences, but is implicitly aligned.  b) Attributes IPV experiences or consequences to structural factors of oppression that align with themes of structural racism and related systems of oppression (e.g., structural violence, legal violence, structural barriers, systemic barriers, fear of deportation, etc.).  (See Table 3, column ‘Key structural factor(s) that align with structural racism’ for specific examples for each article included). |

2. To further strengthen the rigor of your study without contradicting your epistemic stance, please include a specific description of the initial classification discordance and its resolution. As you note, eight articles were initially categorized differently; a single sentence in the Methods or Limitations section will suffice.

We have clarified the description of the initial classification discordance and resolution in lines 266-269.

3. Please copyedit the Discussion and Conclusion sections to address minor textual artifacts, such as spacing issues and typographical errors (for example, “epistemic justicea” and “towarda…ensuringe”).

The Discussion and Conclusion sections have been copyedited.

4. During proofing, please confirm consistency between the PRISMA figure and the text, ensuring that counts and exclusions match exactly throughout.

We have reviewed the manuscript to ensure consistency between the PRISMA figure and the text.

5. For improved readability, it is recommended (though optional) to consider splitting or relocating the densest table content (Tables 2 and 3) to supplementary material, while retaining a concise summary table in the main text.

Thank you for the suggestion. After discussion with the team, we have decided to leave the tables as is. We feel that both tables provide information that many readers will be looking for.
